# Reliability and validity of the Standardized swallowing assessment among community-dwelling older adults in China

**DOI:** 10.1080/07853890.2025.2548980

**Published:** 2025-08-20

**Authors:** Jing Wang, Caixia Chen, Yuzhen Qin, Jing Zeng, Chunhua Zhang, Liugen Wang, Heping Li, Xi Zeng

**Affiliations:** a Zhengzhou University First Affiliated Hospital, Zhengzhou, China; b Zhengzhou University, Zhengzhou, China; c East China Normal University, Shanghai, China; d Guangdong Work Injury Rehabilitation Hospital, Guangzhou, China

**Keywords:** Swallowing, screening, geriatrics, public health

## Abstract

**Background:**

Oropharyngeal Dysphagia (OD) has become a public health issue and early screening has practical significance. The Standardized Swallowing Assessment (SSA) is a clinician-driven, simple, and efficient screening tool but has yet to be validated in Chinese communities.

**Objectives:**

This study aimed to cross-culturally adapt and validate the SSA in Chinese community-dwelling older adults and explore its optimal cut-off value.

**Methods:**

The SSA was cross-culturally adapted according to a 5-stage process. Reliability included internal consistency, inter-rater reliability, and test–retest reliability. Cronbach’s alpha was used to assess its internal consistency. The other reliability analyses were conducted using Pearson’s correlation. Validity analysis included convergent and concurrent validity. For the convergent validity, the correlation between the SSA and Penetration-Aspiration Scale (PAS) or 10-item Eating Assessment Tool (EAT-10) was analyzed using Spearman’s correlation and Mann–Whitney *U* tests. For concurrent validity, the association between the gold standard and the SSA was analyzed using Kruskal–Wallis and Mann–Whitney *U* tests. Receiver Operating Characteristic (ROC) analysis was used to explore the optimal cutoff value.

**Results:**

A total of 466 and 79 Chinese community-dwelling older adults were included in the first and second assessments, respectively. The Cronbach’s coefficients for the total scale and each step were >0.7. The Pearson correlation coefficients were >0.8 for test-retest reliability (*n* = 79) and >0.9 for inter-rater reliability (*n* = 143), indicating excellent temporal stability and consistency across different raters. There were significant correlations between the SSA and both the EAT-10 (*r* > 0.5, *p* < 0.001) and PAS (*r* > 0.4, *p* < 0.001). There were significant differences in the SSA scores between the participants with the EAT-10 ≤ 3 and those with the EAT-10 > 3 (*p* < 0.001), or those with the PAS ≤ 3 and those with the PAS > 2 (*p* < 0.001). The Kruskal-Wallis test showed significant differences in the SSA scores across clinical severity (*H* = 142.388, *p* < 0.001). The optimal cutoff value was found to be 21.0 to distinguish healthy individuals from OD patients (C-index = 0.801, sensitivity = 0.983, specificity = 0.610), and to be 28.0 to differentiate mild OD from moderate-to-severe OD (C-index = 0.875, sensitivity = 0.914, specificity = 0.736).

**Conclusions:**

The SSA showed good reliability and validity among community-dwelling older adults in China, and the optimal cutoff values were 21.0 and 28.0.

## 1. Introduction

The global proportion of the older population has been continuously rising and presenting challenges to society and healthcare systems [1]. As aging progresses, bodily functions gradually decline, and the coordination and strength of swallowing muscles weaken [2]. This increases the risk of dysphagia among older individuals. Many common geriatric conditions are associated with swallowing function, impacting self-care abilities and quality of life [2,3].

Oropharyngeal Dysphagia (OD) refers to swallowing difficulties caused by issues in the muscles or nerves of the oropharyngeal region [4]. It is recognized as a significant health issue, particularly among older population [5]. Mild OD is typically subtle, characterized by slow eating and oral intake limits [6]. As symptoms progress, it increases the risk of various adverse outcomes, including malnutrition, dehydration, and aspiration pneumonia [7]. In addition, OD is associated with psychological disorders and can affect the quality of life of both patients and their families [8,9]. These issues are closely related to healthy aging, and therefore, OD is a public health concern. OD has been reported to affect 11.4% to 33.7% of older adults [10]. This prevalence deserves attention and highlights the importance of early detection. Among older adults, factors such as age-related changes in swallowing physiology, the presence of comorbid conditions, and cognitive decline further complicate the diagnosis and management of OD [11,12]. Consequently, there is an urgent need for reliable and efficient screening tools to identify OD in this vulnerable demographic.

The gold standard for diagnosing OD is the Videofluoroscopic Swallow Study (VFSS) [13]. In regions lacking fluoroscopy equipment, the Fiberoptic Endoscopic Evaluation of Swallowing is also considered highly accurate and reliable [14]. However, both methods are time-consuming, expensive, and require specialized equipment. This limits the widespread use outside hospital or research settings [15]. Some scholars have suggested that individuals could first undergo OD screening, and those with suspected OD can be further assessed using imaging studies [16]. OD screening tools can be categorized into questionnaires and tests [17]. Common OD screening questionnaires include the Sydney Swallow Questionnaire [18], the Swallowing Disturbance Questionnaire [19], and the Eating Assessment Tool (EAT-10), etc. These tools assess the risk of OD through a series of items and users’ subjective responses [16]. Among them, the EAT-10 is the most representative [20]. It has undergone cross-cultural adaptation in many countries and demonstrated overall good validity [16]. While simple and non-invasive, these questionnaire-based tools have been discussed for the lack of objectivity, as the results primarily depend on users’ perceptions and can be influenced by users’ understanding of dysphagia or cognitive states [16]. Swallowing screening tests typically require users to perform a series of movements or behaviors [17]. Common OD screening tests include the 3-ounce water swallow test [21], the Mann Assessment of Swallowing Ability [22], and the Gugging Swallowing Screen [23], etc. These screenings generally involve professionals’ observations and scoring, making them relatively objective [17]. Tools like the 3-ounce water swallow test are convenient and time-efficient but focus only on a single consistency of bolus or a specific aspect of swallowing. Such tools may not comprehensively address all aspects of daily swallowing [24]. The Mann Assessment of Swallowing Ability and Gugging Swallowing Screen are relatively complex, often taking over 15 min to complete [22,25]. These tools show promising potential for clinical applications. However, in community settings, they may lack cost-effectiveness and are less suitable for areas with limited healthcare resources [26]. This gap in the available OD screening tools has created a need for simple, accessible, and accurate methods that can be effectively implemented in diverse healthcare settings.

The Standardized Swallowing Assessment (SSA, the original developers of the SSA have authorized this study) is a clinician-driven screening tool that might serve as an alternative to address the limitations of existing assessments [27]. The SSA involves a set of standardized tasks that can be quickly administered by healthcare staff [27]. It was originally developed as a bedside assessment for stroke patients and has been applied in many other fields, such as traumatic brain injury [28]. The SSA is considered relatively objective as it involves direct observation and clinical judgment rather than relying only on patient-reported symptoms [28]. Additionally, the SSA has no equipment requirements. These characteristics make the SSA potentially valuable tool in community settings. However, its reliability and validity in Chinese community-dwelling older adults have not been reported. The optimal cutoff value for identifying OD risks among this population has yet to be explored. Additionally, although the SSA has been translated into Chinese, it is unclear whether these translations are perfect.

Therefore, this study has three objectives: (1) to cross-culturally adapt the SSA in Chinese context; (2) to assess its reliability and validity among community-dwelling older adults in China; and (3) to explore its optimal cutoff value by analyzing the association between the gold standard and SSA.

## 2. Method

This study was approved by the Ethical Committee of Zhengzhou University First Affiliated Hospital (2024-KY-0708) and was conducted according to the Standards for Reporting Diagnostic Accuracy Studies and the Helsinki Declaration [29]. All participants provided written informed consent.

### 2.1. Study design

This prospective study was conducted in central China in 2024. Participants were required to undergo multiple swallowing function assessments to analyze the reliability and validity of the SSA. Eighty participants were randomly selected to undergo a second assessment 15 days after the first assessment to analyze the test-retest reliability of the SSA. General information was collected at baseline, while assessment results were collected after the evaluation.

### 2.2. Participants

We used convenience sampling to recruit participants from three neighborhoods near the author’s institution in 2024: We posted a brief introduction to this study online and within these neighborhoods. Interested participants can contact the researchers for further information and initial eligibility screens. Participants were consecutively enrolled. Individuals were invited if they were over 60 years old, conscious, able to communicate and read in Chinese, and had lived continuously in mainland China for more than two years. Potential participants were excluded if they had a history of severe mental disorders, hearing comprehension disorders, or had been hospitalized within the past 12 months.

### 2.3. Cultural adaptation of the SSA

We used a five-stage process to culturally adapt the SSA into Chinese [30]. This method has been considered effective in identifying and solving problems in scale translation and has been widely used in cross-cultural studies [31]. We invited ten experts from the fields of rehabilitation medicine, geriatrics, and public health to participate in the translation of the SSA. All of them had been working for at least three years in dysphagia-related fields and had English proficiency of Public English Test-5 or better. The translation process consisted of five steps: (1) These experts independently translated the SSA into Chinese. (2) By reviewing the different translation versions and discussions, these experts resolved linguistic and conceptual issues to reach a consensus version. (3) The consensus version was then translated back into English by three independent translation experts. (4) The ten experts compared the original and the back-translated versions to modify the consensus version and reach the final revision of the SSA. (5) We invited seven doctors, eleven therapists, and eight nurses, all considered potential assessors of the SSA, to read the SSA and use it clinically over a two-week period. They were asked to report any issues they encountered. After this, the final version was approved, as no major adjustments were required, as shown in Appendix 1.

Similar to the original version [27], the Chinese version of the SSA consists of three steps. The total score could range from 18 to 46, with lower scores indicating better swallowing function. For each 2-point item: 1 point indicates normal, and 2 points indicates abnormal. For 3-point or 4-point items: 1–2 points indicate normal, and 3 points or more indicate abnormal. For each step (there are three steps: Step 1 – initial evaluation; Step 2 – drinking 5 ml of water three times; and Step 3 – drinking 60 ml of water): If one item is abnormal, the entire step is considered abnormal. If all items are normal, the entire step is considered normal. If the initial evaluation is abnormal, no further evaluation is needed. The score is calculated as the sum of the scores from the initial evaluation of each item, plus the highest score from Step 2 (11 points) and the highest score from Step 3 (12 points). If the initial evaluation is normal but the Step 2 evaluation is abnormal (i.e. at least two out of three water drinking trials are abnormal), no further Step 3 evaluation is needed. The score is the sum of the scores from the initial evaluation, the scores from Step 2, and the highest score from Step 3 (12 points). If the initial evaluation and Step 2 results are both normal (i.e. at least two out of three water drinking trials are normal), the score is the sum of the scores from each step. Specifically, when all stages are normal, the result is 18 points, indicating normal swallowing function.

### 2.4. Tools for validity

#### 2.4.1. Convergent validity

Convergent validity refers to whether the measurements of the target scale are consistent with scales of similar constructs. We selected both external and patient-reported tools to analyze the convergent validity of the SSA. The first tool was the Penetration-Aspiration Scale (PAS), which has been widely used as an external standard for validation of swallowing-related scales. The PAS was chosen because it can reflect the safety of swallowing [32]. The PAS score could range from 1 to 8, with higher scores indicating poorer airway protection. According to the original developers’ research, the cutoff level for the PAS was set at 2 [33]. The second tool was the 10-item Eating Assessment Tool (EAT-10), which has been cross-culturally adapted into Chinese [34]. As a screening tool for dysphagia, the EAT-10 was considered to share similar purposes with the SSA. However, the EAT-10 is a patient-reported tool rather than a clinician-driven scale. Therefore, it was used to assess the convergent validity of the SSA. The total score could range from 0 to 40, with higher scores indicating worse swallowing function. According to both the original developers’ research and the Chinese adaptation study, the cutoff value for the EAT-10 was set at 3.0 [35,36].

#### 2.4.2. Concurrent validity

Concurrent validity refers to the consistency between the measurements of the target scale and the gold standard. VFSS is considered the gold standard for diagnosing dysphagia, but related standards have not yet been unified [13]. Currently, the Modified Barium Swallowing Impairment Profile (MBSImP) is the most widely recognized VFSS-based swallowing function assessment [37]. This assessment was based on the impressions section of a speech-language therapist, who reviewed the participants’ VFSS recordings. The assessment content included lip closure, tongue movement, oral retention, initiation of pharyngeal swallowing, pharyngeal retention, Upper Esophageal Sphincter opening, and aspiration [37]. To avoid subjective bias, all clinical severity ratings were performed by a single therapist who was certified in the MBSImP. The results included normal, mild, moderate and severe. The standard for the classification is shown in Appendix 2.

### 2.5. Procedure for validation

We trained all staff in advance to minimize potential bias. Before participating in the study, the staff explained the significance, objectives, and outline of the study to the participants who were free to voluntarily complete or withdraw from the study halfway. The staff involved in this validation procedure were speech-language therapists and nurses with >1 year of clinical experience.

After enrollment, we first collected general information from the participants, including sex, education level, and other details. This information was based on identification cards or medical records. If unavailable, it was based on self-reports. Next, the participants underwent the SSA. According to the SSA process, the participants could only proceed to the next SSA step if they were assessed as normal in one SSA step. Otherwise, the assessment ended, and the total score was calculated. Each participant was randomly assigned to an assessor. To analyze the consistency between different assessors, we randomly selected 143 participants. Their SSAs were completed by two assessors, who independently scored the participants without communication during the assessment. After a short break, the participants underwent the EAT-10 assessment. The data were double-entered and uploaded after the staff verified that all items had been completed.

Subsequently, the participants underwent modified barium swallow tests under VFSS. They were instructed to complete the test while seated, without any positional compensation. Contrast agents and thickeners were mixed in varying proportions to create boluses of different consistencies (paste, liquid, and solid). Each consistency was tested three times, and the participants swallowed 5 ml of the bolus each time. If a participant aspirated or penetrated and he/she could not clear the foreign matter independently, no further tests of that consistency were conducted, and safety measures were taken, such as back slapping. The VFSS was conducted at 30 frames per second, including anterior and lateral views. The PAS and clinical severity analyses were independent of each other and based on VFSS. To ensure objectivity, the PAS assessors did not participate in other aspects of this study. Given that community-dwelling individuals may choose various food textures in their daily lives, the worst result from multiple barium swallow tests was used as the basis for the PAS score.

To measure test–retest reliability, we randomly selected 80 participants to undergo the SSA again 15 days after the first assessment. They did not receive any swallowing-related interventions during the 15-day study period.

### 2.6. Sample size

Among various methods, we selected the one that resulted in the largest sample size to ensure the validity and representativeness of this study. To ensure the effectiveness of subgroup analyses [38], we estimated the sample size based on the prevalence of OD. A study indicated that OD affected 39.4% of Chinese community-dwelling older adults [39]. The confidence level, margin of error, and sample loss rate were set at 95%, 5%, and 10%, respectively. The results showed that at least 404 participants were required.

### 2.7. Statistical analysis

Continuous data with skewed distributions and ranked data were presented using medians and quartiles [M (Q_1_, Q_3_)], and categorical data were presented using frequencies and percentages [*n* (%)]. Prior to the statistical analysis, normality and homogeneity of variance tests were conducted.

Reliability analysis included internal consistency, inter-rater reliability, and test-retest reliability. Cronbach’s alpha was used to assess the internal consistency, with a minimum acceptable value of 0.7 [40]. The other reliability analyses were conducted using Pearson’s correlation. Validity analysis included convergent and concurrent validity. For convergent validity, the associations between the SSA and both the PAS and EAT-10 were assessed using Spearman’s correlation and Mann–Whitney *U* tests. For concurrent validity, the participants were divided into three groups based on the MBSImp results. The Kruskal–Wallis test was used to explore the differences among the groups, and Mann–Whitney *U* tests were used for subgroup analysis. To determine the optimal cutoff value, Receiver Operating Characteristic (ROC) analysis was conducted with the MBSImp results (Normal = 0/Other = 1; Mild = 0/Moderate and severe = 1) as the dependent variables and the SSA scores as the independent variable. The optimal cutoff value was the point at which the sum of the accuracy and specificity was maximized [41]. The final analysis included no missing data. A *p*-value < 0.05 was considered significant and SPSS 21.0 was used for statistical analysis.

## 3. Result

### 3.1. General information

Five hundred and thirty-two older adults completed the eligibility screening. There were 6, 10, and 33 excluded cases due to the history of severe mental disorders, hearing comprehension disorders, and hospitalization within 12 months, respectively. A total of 483 participants were included in this study. Among them, 17 cases withdrew from the study due to unwillingness to undergo the VFSS. Ultimately, 466 participants completed the first assessment. One selected participant did not participate in the second assessment. There were 79 participants who completed the second assessment. Based on the PAS results, 390 participants (83.69%) had no risk of airway invasion (The PAS grade < 3). Based on the gold standard of clinical severity, a total of 287 (61.59%), 121 (25.97%), 44 (9.44%), and 14 (3.00%) participants had normal swallowing function, mild OD, moderate OD, and severe OD, respectively. The median completion time for the SSA was 4 (3, 6) minutes. General information of the participants is shown in Table 1.

**Table 1.** General information.

Item	Total participants (*n* = 466)
Sex [*n* (%)]	
Male	181 (38.84)
Female	285 (61.16)
Age [M (Q_1_, Q_3_)]	66 (62, 73)
Education level [*n* (%)]	
Middle school or lower	277 (59.44)
High school or higher	189 (40.56)
History of dysphagia-related diseases [*n* (%)]	
Ischemic stroke	104 (22.32)
Hemorrhagic stroke	21 (4.51)
Traumatic brain injury	34 (7.29)
Neurodegenerative diseases	30 (6.44)
Head and neck cancer	13 (2.79)
Sarcopenia	21 (4.50)
Others	19 (4.08)
Safety of swallowing (according to the Penetration-Aspiration Scale) [*n* (%)]	
Safe (Level 1–2)	390 (83.69)
Unsafe (>Level 2)	176 (16.31)
Severity of dysphagia (according to the Modified Barium Swallowing Impairment Profile) [*n* (%)]	
Normal	287 (61.59)
Mild	121 (25.97)
Moderate	44 (9.44)
Severe	14 (3.00)

### 3.2. Reliability

The internal consistency for all participants was the best, with the Cronbach’s α all greater than 0.8. Considering that some participants did not complete all steps of the SSA, some of the item scores were directly assigned the highest value. Therefore, we assessed internal consistency among participants with different levels of completion. For all groups, both the total score and each step of the SSA had a Cronbach’s α greater than 0.7, as shown in Table 2. This indicated good internal consistency for the SSA. The Pearson correlation coefficient of the SSA total score was 0.941, and the coefficients for each step were all greater than 0.8, as shown in Table 2. This indicated that the SSA results were stable and consistent over time. The inter-rater reliability performed well, with all correlation coefficients greater than 0.9, as shown in Table 2.

**Table 2.** Internal consistency and test–retest reliability.

Item	Cronbach’s α				Test–retest reliability (*n* = 79)	Inter-rater reliability (*n* = 143)
	Participants who completed only Step 1 (*n* = 88)	Participants who completed Steps 1 and 2 (*n* = 145)	Participants who completed the SSA (*n* = 233)	All participants (*n* = 466)	Pearson correlation coefficient	
Total	N/A	0.709	0.855	0.824	0.941***	0.977***
Step 1	0.713	0.737	0.749	0.842	0.877***	0.931***
Step 2	N/A	0.721	0.765	0.902	0.840***	0.940***
Step 3	N/A	N/A	0.787	0.948	0.902***	0.969***

*Notes*: The analyzed variables for the total score were the scores from each step. The analyzed variables for each step were all the items included in that step. SSA Standardized Swallowing Assessment.

*** *p* < 0.001.

### 3.3. Validity

#### 3.3.1. Convergent validity

The Spearman correlation analysis showed a coefficient of 0.437 between the SSA score and PAS grades (*r* = 0.437, *p* < 0.001), and a coefficient of 0.539 between the SSA and EAT-10 scores (*r* = 0.539, *p* < 0.001). The participants were divided into two groups based on the thresholds of the PAS = 2 and the EAT-10 = 3. The Mann–Whitney *U* tests showed significant differences between groups based on the EAT-10 and groups based on the PAS. For the groups based on the EAT-10, the EAT-10 < 3 group showed a SSA score of [20.0 (18.0, 28.0)] while the EAT-10 ≥ 3 group showed a SSA score of [28.0 (26.0, 36.0)], with significant differences (*p* < 0.001). For the groups based on the PAS, the PAS ≤ 2 group showed a SSA score of [25.0 (18.0, 28.0)] while the PAS ≥ 3 group showed a SSA score of [31.0 (26.0, 37.0)], with significant differences (*p* < 0.001).

#### 3.3.2. Concurrent validity

Based on the MBSImp results, the participants were divided into three groups: normal, mild, and moderate-to-severe. The Kruskal–Wallis test showed significant differences in the SSA scores among the three groups (*H* = 142.388, *p* < 0.001). To further analyze the validity of the SSA, Mann–Whitney *U* tests were conducted to examine the differences between subgroups. The results indicated that the SSA can effectively distinguish between normal swallowing function, mild OD, and moderate-to-severe OD, as shown in Table 3.

**Table 3.** Concurrent validity analysis.

Group	SSA scores [M (Q_1_, Q_3_)]	z/H	*p*
Normal (*n* = 287)	19.0 (18.0, 28.0)		
vs. Mild + moderate-to-severe (*n* = 179)	28.0 (26.0, 34.0)	11.809	<0.001***
Mild (*n* = 121)	26.0 (25.0, 29.0)		
vs. Normal (*n* = 287)		7.338	<0.001***
Moderate-to-severe (*n* = 58)	36.0 (30.0, 38.0)		
vs. Mild (*n* = 121)		8.128	<0.001***
vs. Mild + Normal (*n* = 408)	25.0 (18.0, 28.0)	9.994	<0.001***
vs. Mild vs. Normal		142.388	<0.001***

*Notes*: SSA Standardized Swallowing Assessment.

*** *p* < 0.001.

### 3.4. Optimal cutoff value

**Figure 1.**
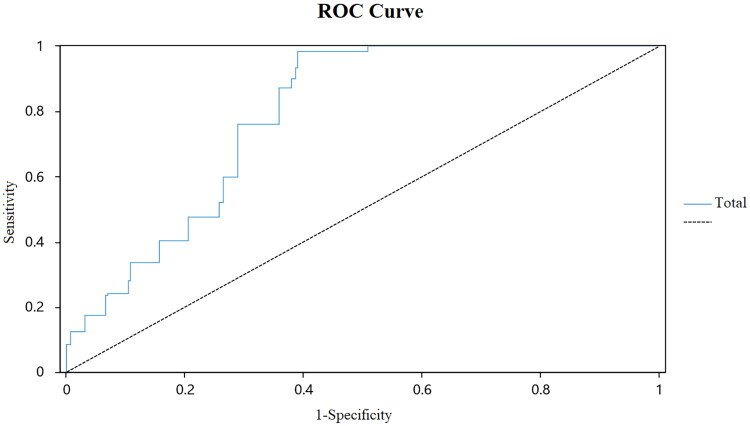
ROC curves 1.

**Figure 2.**
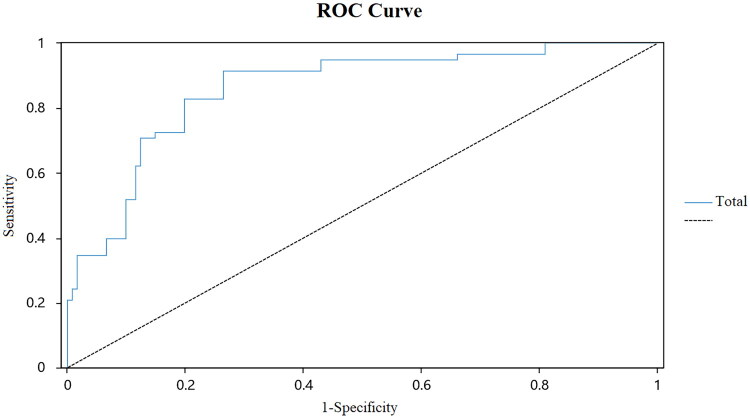
ROC curves 2.

We conducted ROC analysis using the SSA score as the independent variable and the swallowing function (normal or OD, based on the MBSImp) as the dependent variable, as shown in Figure 1. The results showed that the Area Under Curve (AUC) was 0.801 (95% CI: 0.762–0.839), indicating that the SSA has a high diagnostic value for OD. The sensitivity was 0.983, the specificity was 0.610, and the optimal cutoff value was 21.0. To further analyze the ability of the SSA to differentiate mild from moderate-to-severe OD, we performed ROC analysis among OD participants, as shown in Figure 2. The results showed an AUC of 0.875 (95% CI: 0.820–0.929), with a sensitivity of 0.914, specificity of 0.736, and an optimal cutoff value of 28.0. Therefore, the SSA = 21.0 can be used to distinguish healthy individuals from OD patients, whereas the SSA = 28.0 can be used to differentiate mild OD from moderate-to-severe OD.

## 4. Discussion

Swallowing problems in older adults are often overlooked [6]. If early-stage OD progresses to the point of affecting swallowing safety or oral intake amount, necessary enteral nutrition support can further impact psychological well-being and daily living [42]. The SSA is simple, quick to administer, and non-invasive, making it a potential tool for community-based OD screening. The current study conducted rigorous cultural adaptation and validation of the SSA into Chinese. The SSA appears to be a valid and reliable clinician-driven tool to screen OD among older adults in Chinese community settings. This provided a valuable reference for community OD screening with significant practical significance.

The SSA showed good internal consistency, with a Cronbach’s α exceeding 0.7. Notably, Cronbach’s α values were consistently above 0.8 for those who completed all steps, indicating the highest internal consistency within this cohort. The SSA also showed good stability and reproducibility over time. These results are consistent with the SSA studies conducted in South Korean nursing homes and in Chinese stroke units [43,44]. Furthermore, the inter-rater reliability was exceptionally high, with all correlation coefficients exceeding 0.9, indicating strong agreement among different evaluators in the assessment process. The similar conclusion was reached in a Canadian study [28]. The high internal consistency, test-retest and inter-rater reliability, supports the utility of the SSA in both clinical and research settings.

The correlations and significant group differences supported the alignment of the SSA with the established tools. This indicated that the SSA had potential applicability in detecting various levels of swallowing dysfunction. The ANOVA and subgroup analysis showed that the SSA could effectively distinguish between healthy individuals, mild OD, and moderate-to-severe OD. However, we did not assess the ability to differentiate between moderate and severe OD. As a screening tool, the main function of the SSA was considered to identify individuals at risk for OD. In clinical practice, patients with moderate or severe OD are recommended for swallowing interventions and enteral nutritional support [45,46]. These therapies rely on more detailed swallowing assessments than the SSA, such as VFSS or fiberoptic endoscopic evaluation of swallowing.

It should be noted that the Spearman coefficient of the SSA in convergent validity was not particularly strong, with the r between 0.4 and 0.6 falling into the moderate range. Compared to the modified barium swallow study [47], the SSA only instructed participants to repeatedly swallow water. The difference in bolus consistency might have influenced the correlation between the tools. Compared to the SSA, the EAT-10 additionally focuses on tension and pleasure during swallowing, as well as the impact of swallowing on social interactions and body weight [16]. These dimensional differences might have affected the correlation between the tools. It is important to note that attitudes toward Spearman coefficients could vary across disciplines. The EAT-10 involves psychological characteristics, and in psychological research, a correlation coefficient > 0.5 is generally considered acceptable [48]. The SSA showed relatively low correlation coefficients but strong abilities to distinguish between healthy individuals and patients. This might indicate that, rather than quantifying the severity of OD, the SSA may be more suitable for qualitative screening.

This study preliminarily explored the optimal cutoff value of the SSA. Some studies identified that the OD risk was low for individuals who passed the three water trials [43]. Some clinical research reported that the SSA cutoff of 18 and 31 could indicate the OD risks and the need for tube feeding, respectively [49,50]. However, there is still a lack of real-world evidence from the community. In the ROC analysis, the difference between sensitivity and specificity is noteworthy. In both curves, the SSA demonstrated satisfactory sensitivity, while the specificity was slightly lower. A study from Korea showed that the sensitivity of the SSA in OD screening was 0.94, and the specificity was 0.65 [51]. Another observational study indicated that the SSA had a sensitivity and specificity of 92% and 50%, respectively, in patients with traumatic brain injury [28]. However, it has been reported that the specificity and accuracy of the SSA are very high in patients with stroke or cervical spinal cord injury [28,52]. These results are consistent with the current study. This indicated that SSA can effectively identify individuals at high risk of OD as a screening tool but may produce some false positives. This might be a common issue among swallowing screening tools. A review pointed out that screening tools should possess high sensitivity and moderate specificity in order to effectively identify individuals with OD [17]. Some screening tools based on water swallowing tests showed increased sensitivity but decreased specificity as the times of water tested increased [53]. The SSA involves multiple water swallowing tests, which may contribute to its reduced specificity. Therefore, the SSA is recommended as an initial screening tool. Detailed follow-up examinations are necessary to diagnose clinical severity. Moreover, the original study of the EAT-10 mentioned that the EAT-10 can be used together with water swallow tests, which can improve sensitivity and specificity [35]. Therefore, the SSA may be used in combination with the EAT-10 to ensure that the assessment can consider both subjective and objective aspects.

This study had several limitations. First, this was a small-sample study, and the samples were sourced only from central China. Therefore, the conclusions should be cautiously generalized to other populations. Second, the retest interval was chosen based on our clinical experience. Given the relatively stable nature of chronic dysphagia, a longer interval between measurements may be feasible. Third, since moderate-to-severe OD is not common in the community, we did not explore the diagnostic capability of the SSA between moderate and severe OD. Fourth, we have not assessed the reliability and validity of the combined use of the SSA and other patient-reported tools, nor have we included flexible laryngoscopy in the validity analysis. Additionally, the participants were not very old and were cognitively relatively intact so the applicability of the results to older age groups and people with dementia is limited. These issues are to be addressed in future, large-scale cohort studies and meta-analyses.

## 5. Conclusion

In conclusion, the SSA has demonstrated strong reliability and validity as an OD screening tool among community-dwelling older adults in China. The optimal cutoff values were 21.0 to distinguish healthy individuals from OD patients and 28.0 to differentiate mild OD from moderate-to-severe OD. The SSA has broad application prospects in community screening for OD in China.

## Supplementary Material

Appendix.docx

## Data Availability

The datasets generated and analyzed during the current study are not publicly available due to the hospital’s confidentiality regulations regarding trial data but are available from the corresponding author Zeng Xi on a reasonable request.
