# Supplementary material for: Reliability and validity of the Standardized swallowing assessment among community-dwelling older adults in China
Source: Ann Med. 2025 Aug 20;57(1):2548980. doi: 10.1080/07853890.2025.2548980 (PMC12369521; doi:10.1080/07853890.2025.2548980)
Supplement: Appendix.docx [file IANN_A_2548980_SM2632.docx]

Appendix 1. The Chinese Version of the SSA

标准吞咽障碍评估 (Standardized Swallowing Assessment)

SSA第一步 (Step 1) 工作人员检查 (Staff check)

1. 受试者意识水平 (Level of Consciousness)

清醒(Awake , 1分)

嗜睡，可唤醒，可对语言作出反应(Drowsy, can be awakened, responds to speech, 2分)

呼唤有反应，但闭目不语(Responds to calling but remains silent with eyes closed, 3分)

仅对疼痛刺激有反应(Responds only to pain, 4分)

1. 头部与躯干控制 (Head and Trunk Control)

能正常维持坐姿平衡(Can maintain sitting balance normally, 1分)

能维持坐姿平衡但时间短(Can maintain sitting balance but for a short time, 2分)

不能维持坐姿，但能部分控制头部平衡(Cannot maintain sitting balance but can partially control head balance, 3分)

不能控制头部平衡(Cannot control head balance, 4分)

1. 有无呼吸困难 (Dyspnea)

正常(Normal, 1分)

异常 (Abnormal, 2分)

1. 有无流涎 (Drooling)

正常(Normal, 1分)

异常 (Abnormal, 2分)

1. 舌活动是否对称 (Tongue Movement Symmetry)

正常(Normal, 1分)

不对称 (Asymmetrical, 2分)

无法活动 (No movement, 3分)

1. 有无构音障碍、湿性发音、声音嘶哑 (Speech Impairment, Wet Voice, Hoarseness)

无(Normal, 1分)

轻度 (Mild, 2分)

重度 (Severe, 3分)

1. 咽反射是否存在 (Gag Reflex)

正常(Normal, 1分)

异常 (Abnormal, 2分)

1. 自主咳嗽能力 (Ability to Cough Spontaneously)

正常(Normal, 1分)

减弱(Reduced, 2分)

缺乏 (Absent, 3分)

SSA第二步 (Step 2)，观察患者三次各饮5ml水 (Observe the patient drinking 5 ml of water three times)

1. 口角流水 (Mouth Corner Dripping)

0-1次 (0-1 time, 1分)

大于1次 (More than 1 time, 2分)

1. 吞咽动作 (Swallowing Movement)

有(Present, 1分)

无 (Absent, 2分)

11. 重复吞咽 (Repeated Swallowing)

0-1次 (0-1 time, 1分)

大于1次 (More than 1 time, 2分)

1. 吞咽时气促、咳嗽 (Shortness of Breath or Coughing During Swallowing)

无(None, 1分)

有 (Present, 2分)

1. 吞咽后湿性发音、声音嘶哑 (Wet Voice or Hoarseness After Swallowing)

正常(Normal, 1分)

减弱或声音嘶哑(Reduced or hoarse, 2分)

发音不能 (Unable to produce sound, 3分)

患者在第二步中至少两次饮水 全部正常，则观察患者饮60ml水并回答接下来的问题

If the patient drinks 5 ml of water normally in at least two out of three trials in Step 2, observe the patient drinking 60 ml of water and answer the following questions:

1. 能否全部饮完 (Can the patient drink the water up)

是(Yes, 1分)

否 (No, 2分)

1. 吞咽中或吞咽后咳嗽 (Coughing During or After Swallowing)

无(No, 1分)

有 (Yes, 2分)

1. 吞咽中或吞咽后喘气 (Shortness of Breath During or After Swallowing)

无(No, 1分)

有 (Yes, 2分)

1. 吞咽后湿性发音、声音嘶哑 (Wet Voice or Hoarseness After Swallowing)

正常(Normal, 1分)

减弱或声音嘶哑(Reduced or hoarse, 2分)

发音不能 (Unable to produce sound, 3分)

1. 是否存在误吸 (Aspiration )

无(None, 1分)

可能(Possible, 2分)

存在 (Present, 3分)

Appendix 2. The standard for the classification of MBSImP

| Category | MBSImP Impairments | Residue | Aspiration/Penetration | Functional Impact |
| --- | --- | --- | --- | --- |
| Normal | None (0 impairments) | None | None | No functional limitations |
| Mild Dysphagia | 1–5 mild impairments | Minimal, if any | Occasional, if present | Mild functional limitations |
| Moderate-to-Severe Dysphagia | 6+ significant impairments | Severe, consistent | Frequent, severe | Significant functional limitations |
